# Toward Actionable Practice Parameters for “Dual Diagnosis”: Principles of Assessment and Management for Co-Occurring Psychiatric and Intellectual/Developmental Disability

**DOI:** 10.1007/s11920-020-1127-8

**Published:** 2020-02-01

**Authors:** John N. Constantino, Shae Strom, Michael Bunis, Cy Nadler, Teresa Rodgers, Julia LePage, Connie Cahalan, Amber Stockreef, Lucas Evans, Rachel Jones, Alyssa Wilson

**Affiliations:** 1 Department of Psychiatry, Washington University School of Medicine, St. Louis, MO 63110 USA; 2 Children’s Mercy Kansas City, Kansas City, MO USA; 3 Missouri Department of Mental Health, Jefferson City, MO USA; 4 College for Public Health and Social Justice, Saint Louis University, St. Louis, MO USA

**Keywords:** Comorbidity, Psychiatric services, Diagnosis, Treatment

## Abstract

**Purpose of Review:**

Although treatment algorithms and parameters for best practice are readily available for all major syndromes of psychiatric impairment, the occurrence of psychiatric syndromes in individuals with intellectual and developmental disability (IDD) invokes serious contextual challenges for interpretation of symptoms, diagnosis, and optimization of treatment, both for clinicians and for the service sectors in which care and support of individuals with IDD are delivered. Recognizing that there exist very few definitive resources for best practice under the circumstance of this form of “dual diagnosis,” the Missouri Department of Mental Health convened an expert panel to conduct a focused review and synthesis of the relevant scientific literature from which to develop guidance in the form of decision support to clinicians. This article summarizes the findings for three of the most common and impairing clusters of psychiatric symptoms that co-occur with IDD—aggression, depression, and addictions.

**Recent Findings:**

Individuals with IDD are at high risk for the development of psychiatric symptoms (PS), which often manifest uniquely in IDD and for which evidence for effective intervention is steadily accruing.

**Summary:**

Interventions that are commonly implemented in the IDD service sector (e.g., functional communication training and positive behavioral support planning) are capable of mitigating severe behavioral impairment, yet rarely invoked when dual diagnosis patients are seen in the psychiatric service sector. Conversely, state-of-the-art interventions for traumatic stress, pharmacotherapy, and psychotherapy have proven capable of improving behavioral impairments in IDD but are typically restricted to the psychiatric service sector, where there exist significant barriers to access for patients with IDD, including limitations imposed by diagnostic eligibility and practitioner experience. Bridging these gaps in knowledge and clinical capacity across the respective IDD and PS service sectors should be of very high priority in strategizing the care and support of IDD patients with serious co-occurring psychiatric conditions.

## Introduction

Although there have been extensive reviews of the literature on the clinical treatment of individual developmental and psychiatric disorders, there is much less information on the approach to patients with combined intellectual/developmental disabilities (IDD) and psychiatric syndromes (PS). Despite the limited literature on combined presentations, individuals with IDD are at a disproportionately high risk of developing PS [1–7]. The high level of unmet clinical needs of individuals with such comorbidities is compounded by the fragmentation of the municipal systems designated for their care, which are commonly fragmented into separate and nonoverlapping bureaucracies—one for developmental disability and another for behavioral health services. Since patients with dual diagnoses may have severe and persistent impairments in adaptive function (a glossary for definitions of this and other selected terms is provided in Table 1), they are often heavily reliant on governmental systems of care. Lack of knowledge and experience [8], and insufficient clinical infrastructure for patients with multiple disabilities results in less effective services and interventions that too-often fail to meet the needs of individuals with co-occurring IDD and PS.

**Table 1:** Glossary of terms

Glossary	
Adaptive function	“The child’s performance across socialization, communication, and daily living domains” [9]. Deficits in adaptive function may be influenced by symptoms of a condition but differ from symptoms in that they relate to general aspects of maturity and homeostasis that allow an individual to direct the course of his/her own behavior, pursue goals, maintain safety, contribute to the community through work and social interaction, and engage in fulfilling interpersonal relationships
Functional behavior assessment	Involves evaluation of the behavior and of the antecedent and consequences associated with the behavior. An assessment analyzes the precipitants of the behavior and proposes hypotheses about factors that control the behavior. The information gathered guides the intervention by altering conditions so that the desired behaviors are shaped and reinforced [10]
Functional communication training	Functional communication training involves teaching a socially appropriate communicative response that serves the same function as a problem behavior and therefore serves as a substitute for problem behavior. A functional analysis is conducted to identify the environmental events that serve as reinforcers for the problem behavior and the conditions that evoke problem behavior. A socially appropriate communicative response is selected and taught with prompting and a schedule of reinforcement that results in the appropriate response replacing the problem behavior. An example of this would include training a child to say, “help please” when engaged in a difficult task rather than screaming [11•]
Neurotypical	Exhibiting or characteristic of typical neurological development; i.e. pertaining to individuals who are not affected by a neurodevelopmental disorder
Noncontingent reinforcement without extinction	Includes the delivery of a reinforcer on a time-based schedule that does not depend on the individual’s adaptive or maladaptive behavior. For example, noncontingent reinforcement without extinction may involve allowing an individual to access preferred items every 30 s, irrespective of the individual’s behavior, and without any specific contingency for the preferred item that would operate to extinguish a maladaptive behavior [12]

Current understanding of the causal origins of neuropsychiatric disorders reinforces epidemiologic evidence that comorbidity of PS occurs disproportionately among patients with IDD and that these two domains of neuropsychiatric impairment often overlap with respect to traceable molecular genetic causes [9, 10]. The notion of *pleiotropy* implies that specific genetic, environmental, and behavioral liabilities can contribute to the development of disparate disorders that cross the boundaries of what has traditionally divided IDD from PS. This newly established understanding of biological overlap, coupled with overwhelming epidemiologic evidence of the common co-occurrence of IDD and PS, calls into question traditional categorizations (IDD versus PS) for the purpose of intervention and service planning and, as we will elaborate below, typically results in only partial applications of best clinical practice [11•]. For many patients, the development of higher-impact treatment will require joint implementation of interventions traditionally relegated to one domain or the other. Moreover, capitalizing on knowledge of how PS manifests uniquely in IDD and how it can arise from consequences of IDD that are distinct from those in neurotypical individuals (individuals that are not affected with a developmental disorder) with PS may well improve approaches to effective treatment, carrying the potential for (i) preventing enduring or entrenched trajectories of behavioral impairment; (ii) improving quality of life; and (iii) avoiding unnecessary polypharmacy or placement in overly restrictive environments.

The emergence of PS in individuals with IDD poses many serious challenges for interpretation of symptoms, diagnosis, and optimization of treatment, both for clinicians and for the service sectors which deliver care to support such individuals. Fortunately, serious efforts to establish expert consensus on refining the application of psychiatric diagnostic criteria to patients with intellectual disability has resulted in an important resource for clinicians now in its second edition, the *Diagnostic Manual-Intellectual Disability (DM-ID-2)*, published by the National Association for Dual Diagnosis. Recognizing, however, that there exist very few definitive resources for best practice under the circumstance of this form of “dual diagnosis,” the Missouri Department of Mental Health recently convened an expert multidisciplinary panel to conduct a focused review of the relevant scientific literature and to develop guidance in the form of clinical decision support for three of the most common and impairing clusters of psychiatric symptoms that co-occur with IDD—aggression, depression, and the addictions—within the context of two major categories of IDD: intellectual disability, autism spectrum disorder, and their combination. One of the obvious complexities in any attempt to develop assessment and intervention guidelines for dual diagnosis is the wide variation in IDD severity within the population. For example, DSM-5 parameterizes the degree of impairment of autism spectrum disorder (ASD) according to level of necessary support (requiring support, substantial support, or very substantial support). This stratification does not necessarily result in actionable categorizations relating to an individual’s functional communication skills, cognitive/developmental abilities (including presence of intellectual disability, which co-occurs with ASD in about 25% of patients), and daily living skills. Moreover, an individual’s age and developmental level (infancy, early childhood, school age, adolescence, adulthood, senescence) must also be considered in any algorithm for identifying and treating PS.

### Precipitating Factors for Psychiatric Symptoms Complicated by the Context of Developmental Disability

An irony for what are considered “hard-wired” neurodevelopmental disorders is that they render many individuals affected by IDD exquisitely sensitive to changes in the environment. Abrupt changes in social, residential, or caregiving environments are common precipitants for the emergence of PS among individuals with IDD. These precipitants can generate a disruption in routine, induce a change in access to rewards in the environment, produce a feeling of loss when a caregiver or beloved staff member rotates off service, and disrupt nuances in stimulus characteristics that may be idiosyncratically aversive or rewarding and therefore highly influential. PS can also arise in the context of an individual’s first recognition of his/her own differences from peers, including identification of skill deficits related to vulnerability. These may include deficits in assertiveness, communication, social competence, and/or educational achievement. Negative peer interactions have been shown to exert independent adverse effects, especially on children with high-functioning ASD who are commonly bullied, teased, or ostracized in educational settings. Social affiliation may be further compromised if an affected individual perseverates or is prone to intense absorption with repetitive behaviors or internal preoccupation and when peers and caregivers are unsure of how to promote the individual’s social engagement and inclusion. Exhaustion or the unmet mental health needs of parents and caregivers can have profound effects on the behavior of individuals with IDD. In all of these scenarios and as in the customary approach to adjustment disorders in psychiatric practice, attention to the environment is critical. Restoring the support previously derived from the environment is a *first principle* in the care of individuals with IDD who experience or manifest new psychiatric symptoms, even when those symptoms appear disproportionate to the expected impact of the antecedent environmental change. There may be times when it is difficult or impossible to restore previous levels of support for individuals with IDD, such as the loss of a caregiver or a required change in placement. In these instances, a new baseline for the individual’s behavior should be established, and time to adjust to a new routine or environment should be afforded prior to a significant change in treatment. Premature implementation of pharmacotherapy or specific behavior intervention in this context can be inefficient and off-target in relation to the origin of the presenting symptoms.

### Unique Considerations of Trauma in IDD

Similarly, trauma is a common causal factor in the emergence of “phenocopies” of psychiatric syndromes occurring in patients with IDD in whom traumatic events may have gone unrecognized for years due to the victims’ inability to articulate memories or any accounts of their occurrence. A global paradigm shift is occurring that embraces a trauma-informed approach based on more than two decades of research showing a strong correlation between ACEs (Adverse Childhood Experiences) and long-term health and social outcomes. Specifically, studies have estimated that having a chronic health condition, including an IDD, is associated with having an 83% higher likelihood of a child experiencing 2 or more ACEs and 73% higher likelihood of experiencing 3 or more ACEs by the age of five [12]. Externalizing behavior, which has a greater incidence among children with IDD, can be related to an increased risk of physical abuse, whereas internalizing behavior or communication and learning problems may be related to increased risk of sexual abuse [13, 14]. Even individuals with subclinical traits of ASD are substantially more commonly victimized by physical, sexual, emotional abuse, or neglect than those without these traits in the general population [15]. During adolescence, the sexual development of children with IDD is often misunderstood or stereotyped as asexual or hypersexual which may increase the risk of exposure to sexual abuse. According to a study conducted by the National Survey of Children’s Health (NSCH), children with IDD are bullied 1.5 to 2 times more than their peers without IDD [16]. Additionally, children with IDD are exposed to traumatizing incidents of physical restraint and seclusion which are not typically experienced by their peers without IDD [17]. Emotional neglect, serious injury, property crime, out-of-home placements, and disrupted treatment are other examples of traumatic events disproportionately experienced by children with IDD. Children and youth with IDD are up to 4 times more likely to witness family domestic violence in comparison to controls [18]. According to a study on the prevalence of various types of ACEs experienced by children with IDD, the most frequent were parental separation/divorce (63.8%), parental mental health problems (33.3%) and to have witnessed violence against a parent (28.9%) [19].

In the same way that the presence of IDD may lead service providers to minimize or overlook the presence of PS, the impact of traumatic experiences can be similarly discounted for individuals with IDD [20, 21]. Children and youth with co-occurring IDD and PS who are admitted to inpatient acute care are at significant risk for long-term institutionalization [22]. Recognition of traumatic experiences requires a high level of vigilance on the part of clinicians, given that patients with IDD may struggle to directly communicate traumatic experiences and because events commonly construed as relatively trivial can be experienced as traumatic by the individual. Clinicians trained to diagnose posttraumatic stress disorder (PTSD), as originally defined in the Diagnostic and Statistical Manual for Mental Disorders (DSM) for an adult population such as combat veterans and burn victims, may not readily recognize the clinical symptoms of PTSD in traumatized individuals with IDD. Symptom expression tends to be complicated by cognitive, affective, social, or physical differences [23]. Since communication is often hindered in this population, it is important to observe changes in behavior and function in considering PTSD: hypervigilance or flashbacks can manifest as agitation; autonomic hyperarousal as overt aggression, disruptive behavior, or self-harm; and nightmares as unexplained sleep problems [21]. It is for these reasons that providers working with children and youth with co-occurring IDD and PS should apply a trauma-informed perspective to aid in diagnosis, treatment planning, and building protective factors for affected individuals and their families.

The impact of trauma on individuals with IDD varies greatly depending on developmental level, which influences the manner in which patients estimate external danger and perceived threats, as well as their own ability to protect themselves and maintain safety [23]. Threat perception varies widely in individuals with IDD and engenders thoughts, feelings, and behaviors that can be difficult for caregivers to predict, interpret, or understand. Perceived threats can relate to heightened negative attributional biases to facial expressions or tone of voice, or to fear of separation from loved ones or valued belongings, or to concerns about somatic symptoms or body integrity, any of which can be specifically exacerbated by various forms of traumatic life experience. Fear and shame may prevent families from reporting private and generational trauma or household dysfunction. Families of children with IDD may also discount trauma reactions and PS and fail to recognize the impact that traumatic stress has on the individual or family system. Other factors that influence family functioning and reporting of trauma are caregiver feelings of inadequacy, grief and loss, differing opinions about parenting techniques, anticipatory anxiety related to safety of an individual with IDD, stress and strain on relationships, and caregiver burnout [24].

When traumatic stress underlies PS for an individual with IDD, supports should incorporate a variety of methods including a focus on principles of positive identity development (self-identity, self-efficacy) and efforts to build social-emotional learning skills such as self-awareness (identifying emotions, accurate self-perception, recognizing strengths, self-confidence, self-efficacy); an emphasis on self-management (impulse control, stress management, self-discipline, self-motivation, goal setting, organizational skills); instruction for social awareness (perspective-taking, empathy, appreciating diversity, respect for others); development of relationship skills (communication, social engagement, relationship building, teamwork); and guidance toward responsible decision-making (identifying problems, analyzing situations, solving problems, evaluating, reflecting, ethical responsibility)—all with the goals of promoting resiliency and preventing future traumatic experiences [25]. All individuals may benefit when care-providers slow down their speech, use language for the individual to understand, present information one point at a time, take frequent pauses to check comprehension, use multisensory input, make specific suggestions for change, allow the individual some time to practice new skills, avoid assumptions that the presented information will generalize to other settings, and include multiple caregivers across various environments [26]. Building family protective factors is also key. This can be accomplished by providing support to families that include asking and answering caregiver questions, providing timely information, addressing traumatic caregiver experiences, promoting secure attachment, promoting a healing and protective environment, fostering family-informed, person-centered planning, helping families access trauma-related supports, partnering with parents to create a recovery plan, and helping families navigate the challenges of systems of care [24]. A trauma-informed approach focuses on safety, trustworthiness, choice, collaboration, empowerment, and cultural competence. Applying these principles when working with individuals with co-occurring disorders has been shown to foster resilience and recovery [27].

## Aggression

Rates of aggressive behavior are higher in individuals with IDD than in typically developing peers. Furthermore, the aggressive behaviors can be more severe, more unpredictable, and more treatment resistant than in patients without IDD [28, 29]. Aggression in IDD is typically reactive rather than predatory in nature, but this is not always the case [30]. Aggression in IDD has been shown to be associated with erosion in social relationships, exhaustion of social support, placement in restrictive school or residential settings, physical intervention, increased risk of being bullied or victimized, increased stress levels in caregivers, and decreased quality of life in both the affected individual and his/her family [2, 31•]. Because changes in an individual’s environment or routine are common precipitants for aggression, it is also important to be aware of any significant change with respect to housing, caregivers, school, or family system impacting the individual. Another common but often overlooked factor related to aggressive behavior in co-occurring IDD and PS is the inability to effectively communicate wants and needs. For such patients, *functional communication training* is an indispensable component of appropriate clinical care [32–34]. Functional communication training involves teaching a socially appropriate communicative response that serves the same function as a problem behavior and therefore serves as a substitute for problem behavior. A functional analysis is conducted to identify the environmental events that serve as reinforcers for the problem behavior and the conditions that evoke problem behavior. A socially appropriate communicative response is selected and taught with prompting and a schedule of reinforcement that results in the appropriate response replacing the problem behavior [32].

In other individuals, aggression is used as a *means* of communication, for example, to communicate frustration or aversion to the environment—in this sense, it takes on a functional (rather than primarily affective) quality for which it is important to invoke *positive behavior support* strategies designed to reduce and replace negative behaviors. Neither functional communication training nor positive behavior support approaches are traditionally prioritized as elements of training in psychiatry or mental health counseling. Positive behavior support (PBS) planning begins by conducting a functional behavior assessment (FBA), which provides in-depth information on the function of problematic behaviors, how they are naturally reinforced, and the constellation of experiences that are motivating to a patient [35]. This information can be incorporated into reward schedules that sustain attempts to replace or help the patient avoid engaging in the target behaviors. FBAs may include an exposure assessment that identifies contextual variables that trigger and maintain the aggression and/or impede socially appropriate behavior. Treatment should focus on reorganizing the environment to proactively minimize the probability of aggression and increase the probability of alternative/socially appropriate responses through both antecedent (events that occur before aggression) manipulation, consequence manipulation, and positive reinforcement strategies [2]. Noncontingent reinforcement without extinction can be utilized to decrease problem behaviors that are unintentionally maintained by social positive reinforcement [36, 37]. Mindfulness strategies can be incorporated into PBS plans to further reduce aggression in patients with dual diagnoses, enabling parents and other caregivers to reduce psychological stress while helping the patients themselves self-manage challenging behaviors through PBS. A 40-week study by Singh et al. [38] evaluated adolescents with ASD (*n* = 47) or with IDD (*n* = 45) and their parents. Results showed significant reductions in aggression and disruptive behavior and increases in compliance behaviors in the adolescents in both groups and suggested that the program may be effective regardless of baseline levels of parental stress.

Medical conditions that incur pain and may not be communicated by a patient with IDD should always be considered in the differential diagnosis when aggression emerges in patients with IDD. These include gastrointestinal (GI) distress due to constipation or gastro-esophageal reflux, headaches, and sleep problems including sleep apnea, dental problems, and menstrual pain. Other medical conditions to consider include thyroid function abnormality and the possibility of epilepsy syndromes (particularly temporal lobe epilepsy) for which patients with IDD are disproportionately liable. Untreated medical conditions in individuals with IDD may result in increased problem behaviors and could lead to overreliance on psychiatric medications to control symptoms. In a very recent case report, a child with Down syndrome was misdiagnosed and was prescribed numerous psychiatric medications (clonazepam, venlafaxine, lamotrigine, ziprasidone, and diazepam) over years of time to control aggressive behaviors that ultimately resolved when it was discovered that he had Celiac disease (which disproportionately affects individuals with Down syndrome) and he was placed on an appropriate diet [39]. He subsequently lost 40 pounds that he had gained from the psychiatric medications, his GI symptoms improved, and his behavior improved dramatically.

Psychopharmacologic approaches to the management of aggression in individuals with IDD and PS are an important tool in the treatment of many patients but should be prescribed only after consideration of potential environmental precipitants, preceding or ongoing trauma, needs for functional communication training, and behavior support planning have been carefully considered. Typically pharmacological treatment proceeds similarly as it would for aggression in psychiatric syndromes exclusive of IDD; mood stabilizing agents (lithium, atypical neuroleptics, and anticonvulsant mood stabilizers) are most effective but carry a diversity of short and long term risks. Consequently, it may be prudent to consider stimulants and alpha agonists as first approaches, especially in IDD syndromes in which aggression may be attributable to ADHD or unspecified impulse control problems; this is particularly common in the autism spectrum disorders and represents a significant opportunity for safe, effective amelioration of disruptive behaviors. Anxiolytics can be considered for patients whose aggressive episodes occur in the context of unresolved anxiety, with the caveat that drugs in this class can be disinhibiting and thereby exacerbate aggression in some patients, requiring appropriate caution. There is extensive literature including multiple randomized controlled trials, suggesting that risperidone and aripiprazole have the strongest evidence in reducing aggression and irritability in IDD [40]. These interventions can be combined with parent training and other relevant psychosocial interventions to particular advantage [41, 42]. For ASD patients who have not responded to other antipsychotics, clozapine has been reported to be an efficacious and well-tolerated treatment [43]. As IDD syndromes are becoming increasingly specified by genetic origin through clinical genomic screening, more specified approaches to aggression in particular IDD syndromes are becoming possible, as in the case of Fragile X Syndrome, where several agents have been found to be effective [44], and new clinical trials involving glutamate receptor antagonists and other agents targeting molecular pathways affected by the condition are under way.

## Depression

Depression is common among adolescents and adults with IDD. Pooled estimation of (a) current and (b) lifetime prevalence for depressive disorder among adults with ASD are (a) 23% and (b) 37% respectively, and the 2-year incidence of depression in adults with intellectual disability is on the order of 7.2% [1, 45•]. Whether or not an individual has co-occurring IDD, depression symptoms may be associated with suicidal ideation and/or self-harming behavior, and it is particularly important *in all cases to ascertain whether there is a family history of attempted or completed suicide* given that suicidality is heritable (runs in families) in a manner that is partially independent from depression itself [46]. When depressive symptoms are accompanied by persistent self-harming behaviors in a patient with IDD, specific positive behavior support algorithms can be applied with exhaustive appraisal precipitating biological and environmental factors and to manage these behaviors in accordance with their respective origins [47•].

Common precipitants of depression in IDD are fundamentally similar to those that incur depression in the general population but also have unique features. Loss of a close loved one is one of the most influential causes of depressive symptoms in all people; in IDD, *perceived* loss of a caregiver, friend, or acquaintance who has occupied the *role* of a close loved one can be as salient as the death of a relative. The social and cognitive impairments of an IDD can amplify the perceived salience or importance of a relationship or can result in misinterpretations of natural life events affecting a relationship (a move, a rebuke, a schedule change). In this context, “loss” includes the extent to which disruption of a relied-upon source of interpersonal connection or a sudden increase in social marginalization has led to a greater degree of “disconnection” from the family, community, society, or social group. If such an event antedates the onset of depression symptoms, the treatment approach should reflect what would be invoked for *complicated bereavement* (a specific type of adjustment disorder) and should consider opportunities for “surrogacy” (i.e., to succeed the individual who was lost).

Individuals with IDD can also experience existential crises that comprise a set of specific adjustment disorders commonly encountered during adolescence in neurotypical individuals; these impinge upon the complex and deeply personal structure by which a disability has been incorporated into identity, and can often be associated with depressed mood [48, 49]. The themes of existential crises tend to revolve around concerns pertaining to *mortality* (fear of death or denial of death by risk-taking), *meaninglessness* (concern that limitations of one’s ability severely jeopardize meaning or purpose), *isolation*, or the perceived inability to make a *choice*. The latter refers to situations in which *agency*, or the capacity to direct one’s own course has been violated (the notion of being controlled or “managed” as if a puppet). It is common for individuals with IDD to encounter circumstances in which limitation, marginalization, or loss of control dominate day-to-day experience. When such experiences play a role in precipitating depressive states, the intervention approach must include addressing the existential concern being experienced by the individual in an age- and developmentally appropriate manner and avoiding the assumption that the individual is incapable of formulating or struggling with such concerns.

Peers and the environment fundamentally shape self-perception and the manner in which a disability is incorporated into an individual’s identity. Many children and adolescents with IDD recognize differences between themselves and their peers, and this recognition can be correlated with higher levels of depressive symptoms. Verbally fluent youth with autism who perceived that they had the lowest group membership and social integration have been reported to have elevated levels of depressive symptoms, and these represent targetable precipitants in strategies to treat depression in IDD [50, 51]. Negative peer interactions have been shown to exert independent adverse effects, especially on children with IDD who are very commonly bullied, teased, or ostracized in educational settings. Peer victimization and depressive symptomatology have been widely correlated, and observational studies document both social isolation and distress as direct consequences of being bullied in the school environment. Interpersonal conflict with family members has also been strongly associated with depressive symptomatology. Social marginalization and sedentary lifestyles compound liability for depression and must be addressed in any comprehensive approach to the treatment of depression including those with a dual diagnosis. Young adults with ASD without an intellectual disability are 3 times more likely to have “no daytime activities” compared with adults with ASD *with* an intellectual disability, demonstrating how inadequacy of current service systems to meet the needs of autistic youth without IDD compounds risk for the development/entrenchment of depressive syndromes.

The approach to management of depression in IDD hinges on specification of precipitating factors and careful consideration in ruling out potential medical contributors such as hypothyroidism, chronic pain, epilepsy, insomnia, and catatonia syndromes that are common to some IDDs such as Down syndrome. Catatonia in Down syndrome (which involves social withdrawal) has been responsive to interventions that would *not* necessarily be considered as first-line therapy for patients manifesting depression—such interventions include judicious use of lorazepam, electroconvulsive therapy, and glutamate antagonists [52]. Intervention must be tailored to the individual’s verbal abilities, developmental level, interests, and goals. It is important to recognize that *higher* verbal/cognitive abilities and greater social insight are associated with lower self-perceived social competence and subsequently higher rates of depression [53]. Individuals with IDD and depression benefit from environmental supports to mitigate the impact of precipitating experiences and improve social connectedness and self-determination. A recent review in this journal summarized the literature on psychosocial treatments for anxiety and depression in autism spectrum disorder, highlighting established efficacy of cognitive behavioral therapy (CBT) for anxiety, but few studies have examined the effect of the intervention for depression per se [54•]. There are few studies of good quality evaluating non-pharmacological interventions for adults with IDD and depression; however, the existing studies on CBT show good results in decreasing depressive symptoms [55•]. Additionally, behavioral activation (prescribed engagement in productive activities) with guided self-help treatments has been found effective in reducing depressive symptomatology [56]. This aligns with the literature on behavioral activation as a key mechanism in many CBT programs for individuals with and without IDD [57]. Vocational rehabilitation can be a key support for adults whose depressive symptoms relate to social marginalization, a sense of meaninglessness of daily routines (an existential concern), and/or absence of opportunity to participate meaningfully in the community.

Psychopharmacology and other somatic therapies may be beneficial in treating depression for individuals with IDD, although data are limited. Extrapolation of published effects of antidepressant medications in the psychiatry literature justifies psychopharmacologic treatment for dual diagnosis patients when the modalities described above have been considered and/or failed. Psychopharmacology of depression in IDD is similar to that implemented for major depression in neurotypical individuals, with the exception of dose-adjustments for conditions associated with abnormal drug metabolism or sensitivity to adverse effects. Studies of the efficacy of the various classes of antidepressant medication in IDD are sparse and in many cases limited to case series, but use of these approaches is both reasonable and potentially life-saving when psychosocial strategies have been unsuccessful in reducing severe depression symptoms. Medication classes for treatment of depression include the antidepressants (SSRI’s, SNRI’s, tricyclics, MAO inhibitors, and bupropion), as well as mood stabilizers for adjunctive therapy in treatment-resistant depression (lithium, neuroleptic mood stabilizers, and the anticonvulsants).

## The Addictions

Similar to neurotypical peers, individuals with IDD often start explorative substance use in adolescence (between ages 14–16) yet exhibit lower tolerance for recreational substance use and are at a heightened risk for adverse consequences depending upon intellectual, social, and communicative levels of functioning [3, 58]. It is important to understand the spectrum of substance activity/use, which ranges from recreational and non-problematic use to at-risk/problematic use to severe misuse/dependency. Recreational and explorative use will present differently than risky or problematic and harmful misuse. Furthermore, contextual specifics (such as age or cognitive functioning level) are important to consider when determining severity of substance activity/use, especially in scenarios where access to illicit drugs constitutes a form of neglect or endangerment by the caregiving environment. In addition to substance abuse, excessive and problematic engagement in other specific behaviors (e.g., video gaming, pornography) are included within the addictions umbrella; although detailed coverage of these conditions is beyond the scope of this review, they overlap in many aspects of phenomenology and in psychosocial approaches to treatment [59, 60].

The assessment of patterns of substance use in individuals with IDD requires systematic review of access, safety concerns, and respect for autonomy and decision-making. Typically it is important to clinically ascertain what happens before—e.g., boredom versus withdrawal symptomatology versus co-occurring activities where other people are engaging in substance activity/use—and after substance activity/use, including physiological (e.g., “high” or “drunk” or arousal), social (e.g., social isolation or high-quality social interaction), and psychological (e.g., emotional/mood swings) effects. Immediate and delayed positive and negative consequences of substance activity/use should be reviewed.

Use of harm-reduction strategies or reduction of use over time constitute the prevailing strategies for treating clients with IDD and substance use disorders [58]. When considering harm-reduction strategies, clinicians must target frequency of use, quantity (or amount of time engaged in activity) of use, duration of time between use, and the safety of the environment (e.g., absolute avoidance of driving while intoxicated, restricting recreational substance use to circumstances/settings that minimize risk for predation, exploitation, or self-endangerment in the context of use). Service providers should consider cross-training staff/caregivers on risks and vulnerabilities of clients with IDD, including peer pressure and exploitation, deficits in self-determination, and aspects of autonomy in recreational use. Despite substantial rates of addiction among adolescents and adults with IDD, evidence for the effectiveness of specific therapies varies, and the field is still maturing. There is emerging evidence that modified versions of CBT and motivational interviewing are capable of decreasing aspects of substance activity/use [61•, 62]. Clinicians providing CBT to patients with IDD should be aware of modifications that have improved its application to this subpopulation, and monthly group supervision has been helpful in implementing such modifications [61], which include enhancement in structure, directive strategies, increased number of sessions, and adaptations to meet the communication and comprehension needs of individual clients.

Functional behavior assessments and corresponding behavioral intervention plans have also been shown to be effective at decreasing substance activity/use and can be useful approaches for IDD patients struggling with addiction. To minimize aversive experiences of detoxification (for substance use) and withdrawal (for substance and activity use), treatment should target small successive reductions of substance activity/use over time (i.e., harm reduction), with a long-term goal of abstinence or controlled/responsible use. Goals and timeline to abstinence of substance activity/use should be client-specific and should operationalize harmful levels of use at the beginning of treatment. Group treatment for substance use, such as “12-step” programs, lacks strong evidence for individuals with IDD but can be considered on a case-by-case basis. Mandated participation in group sessions may provoke anxiety or anger among individuals with IDD, leading to dropout, rejection by other group members, and induced feelings of failure and exclusion [3]. Motivational enhancement therapy incorporating principles of CBT has shown promise for individuals with IDD experiencing problems related to alcohol misuse: Kouimtsidis et al. conducted a randomized controlled trial of extended brief intervention among subjects whose drinking exceeded a threshold for safety; at 12 weeks following the intervention, the proportion of participants with harmful drinking had decreased by 66.7% and 46.7% for the intervention and the control groups, respectively [63].

## Conclusions

Our review identified a number of recent translational advances in the management of specific psychiatric syndromes in individuals with autism—fewer in those with intellectual disability—and very few scientific articles in which comprehensive approaches to symptom identification/interpretation, diagnosis, and treatment of specific combinations IDD and PS were implemented and evaluated. The evidence base summarized here emphasizes the importance of tailoring the management of psychiatric syndromes to the unique context of a given intellectual or developmental disability, considering the widely varying consequences of such conditions on capacity for communication and engagement in intervention, the function of behavioral impairment, and the manner in which psychiatric symptoms manifest. Table 2 provides a listing of selected clinical trials and systematic reviews, published over the past 5 years, documenting evidence for specific intervention modalities for ASD/ID and aggression, depression, or addictions. Symptom identification must include careful consideration of precipitating factors for psychiatric symptoms that are accentuated in the context of developmental disability and the increased vulnerability to and impact of trauma. Factors that may be less easily communicated by IDD populations must be considered in diagnostic formulation, including traumatic experiences that may be viewed by the community as relatively trivial, medical conditions that incur pain, and the recognition of differences between individuals with IDD and their peers. Intervention strategies must cross the boundaries of what are typically applied to IDD versus PS, using comprehensive approaches such as positive behavior support planning, functional behavior assessments, applied behavior analysis-based interventions, cognitive behavioral therapy, in-home therapeutic support, case management, and psychopharmacologic therapy. It is clear that more research is needed for understanding the impact of treatment and improving best practice in these common complex syndromes, as represented by combinations of IDD with three common, serious behavioral syndromes covered in relative detail here—aggression, depression, and addictions. As we await advances in evidence-informed approaches to these conditions, bridging gaps in knowledge across the respective IDD and PS service sectors should be of very high priority to empower clinicians, patients, and their families to seek treatments that cross traditional bureaucracies of care and integrate disparate intervention modalities (including positive behavior supports, pharmacotherapy, appropriately modified psychotherapy, and trauma-informed care). These intervention modalities should be advocated for implementation within the treatment plans for individual patients whose complex conditions are unnecessarily protracted or aggravated when there exists unrecognized opportunity for better treatment outcome, i.e., when comprehensive approaches harnessing all available evidence-informed interventions have not yet been considered and applied.

**Table 2:** Listing of selected clinical trials and systematic reviews, publication dates 2014–2019, documenting evidence for specific intervention modalities for ASD/ID and aggression, depression, or addictions

Title	Lead author; Year; Citation number in reference list	Intervention modalities
IDD and aggression		
Aggression in autism spectrum disorder: presentation and treatment options	Fitzpatrick et al. *Neuropsychiatric Disease and Treatment* 2016 [2]	-Functional behavioral assessment -Reinforcement strategies -Functional communication training
Shaping complex functional communication responses	Ghaemmaghami et al. *Journal of Applied Behavior Analysis* 2018 [36]	-Shaping -Functional communication training -Complex functional communication responses
Noncontingent reinforcement without extinction plus differential reinforcement of alternative behavior during treatment of problem behavior	Fritz et al. *Journal of Applied Behavior Analysis* 2017 [12]	-Noncontingent reinforcement without extinction -Differential reinforcement of alternative behavior
Meta-analysis of noncontingent reinforcement effects on problem behavior	Richman, et.al, *Journal of Applied Behavior Analysi*s 2015 [38]	-Positive behavior support planning
Effects of mindfulness-based positive behavior support (MBPBS) training are equally beneficial for mothers and their children with autism spectrum disorder or with intellectual disabilities	Singh et al. *Frontiers in Psychology* 2019 [39]	-Mindfulness to reduce perceived psychological stress for both caregivers and children with IDD -Positive behavior support
Pharmacologic treatment of severe irritability and problem behaviors in autism: a systematic review and meta-analysis	Fung et al. *Pediatrics* 2016 [41]	-Risperidone -Aripiprazole
Effect of parent training vs parent education on behavioral problems in children with autism spectrum disorder: a randomized clinical trial	Bearss et al., *JAMA* 2015 [42]	-Behavioral parent training
11 years of clozapine experience in autism spectrum disorder: efficacy and tolerance	Rothärmel et al. *J Clin Psychopharmacol* 2018 [43]	-Clozapine
Pharmacologic interventions for irritability, aggression, agitation, and self-injurious behavior in Fragile X Syndrome: an initial cross-sectional analysis	Eckert et al. *J Autism Dev Disord* 2019 [44]	-Antipsychotic medications, specifically aripiprazole and risperidone
IDD and Depression		
Multidisciplinary assessment and treatment of self-injurious behavior in autism spectrum disorder and intellectual disability: integration of psychological and biological theory and approach	Minshawi et al. *J Autism Dev Disord* 2015 [47^•^]	-Applied behavior analysis (ABA)-based positive behavior supports -Psychopharmacologic intervention
Catatonia in Down syndrome: systematic approach to diagnosis, treatment and outcome assessment based on a case series of seven patients	Miles JH et al. *Neuropsychiatr Dis Treat* 2019 [52]	-Pharmacotherapy and electroconvulsive therapy (ECT)
Non-pharmacological interventions for adults with intellectual disabilities and depression: a systematic review	Hamers et al. *Journal of Intellectual Disability Research* 2018 [55]	-Cognitive behavioral therapy -Behavioral therapy -Exercise intervention -Social problem-solving skills program -Bright light therapy
Comparison of behavioral activation with guided self-help for treatment of depression in adults with intellectual disabilities: a randomized controlled trial	Jahoda et al. *Lancet Psychiatry* 2017 [56]	-Individual psychological interventions: BeatIt and StepUp
Adapting cognitive behavioral techniques to address anxiety and depression in cognitively able emerging adults on the autism spectrum	Kerns et al. *Cognitive and Behavioral Practice* 2016 [57]	-Cognitive behavioral therapy
IDD and addictions		
Acceptance and commitment therapy for problematic internet pornography use: a randomized trial	Crosby et al. *Behavior Therapy*. 2016 [60]	-Acceptance and commitment therapy
Efficacy of short-term treatment of internet and computer game addiction: a randomized clinical trial	Wölfling et al. *JAMA Psychiatry* 2019 [59]	-Short-term, manualized cognitive behavioral therapy, specifically adaptedfor internet/computer game addiction
Treating patients with co-occurring autism spectrum disorder and substance use disorder: a clinical explorative study	Helverschou et al. *Substance Abuse: Research and Treatment* 2019 [61]	-Cognitive behavioral therapy -Monthly ASD education and group supervision to therapists in substance use clinics
A feasibility randomized controlled trial of extended brief intervention for alcohol misuse in adults with mild to moderate intellectual disabilities living in the community; the EBI-LD study	Kouimtsidis et al. *Trials* 2017 [63]	-Manualized motivational enhancement therapy incorporating principles of CBT
